# COVID-19 infection in pediatric subjects: study of 36 cases in Conakry

**DOI:** 10.11604/pamj.supp.2020.37.1.26573

**Published:** 2020-12-08

**Authors:** Hugues Ghislain Atakla, Mahugnon Maurel Ulrich Dénis Noudohounsi, Aichat Yabo Salami, Hélène Sacca, Axel Gaël Houinato, Mamadou Ciré Barry, Guelngar Carlos Othon, Ayeratou Abèbi Adjadi, Dismand Stephan Houinato

**Affiliations:** 1 Neurology Department, University Hospital Center Hubert Koutoukou MAGA, Cotonou, Benin,; 2 Laboratory of Noncommunicable and Neurologic Diseases Epidemiology, Faculty of Health Science, University of Abomey-Calavi, Cotonou, Benin,; 3 Monitoring and Evaluation/WHO AFRO, Research Project Manager, Brazzaville, Congo,; 4 Microbiology Laboratory, University Hospital Center Hubert Koutoukou MAGA, Cotonou, Benin,; 5 Pediatric Department, Ignace Deen University Hospital Center, Conakry, Guinea

**Keywords:** SARS-CoV-2, RT-PCR, pediatric, Conakry

## Abstract

The aim of this study was to evaluate the main clinical and evolutionary features of SARS-CoV-2 infection in children aged 0-18 years who were suspected and diagnosed for COVID-19 during routine consultations in the pediatric ward of the Ignace Deen National Hospital in Conakry. This retrospective study targeted all children admitted to the Pediatrics Department during the study period and focused on children whose clinical examination and/or history indicated a suspicion of SARS-CoV-2 infection. Only children with a positive reverse transcriptase-polymerase chain reaction (RT-PCR) test were included. Clinical and paraclinical data were rigorously analyzed. Anonymity and respect for ethical rules were the norm. Medical records were used as the data source and a questionnaire was developed for collection. The analysis was done using STATA/SE version 11.2 software. The mean age of the patients observed was 9.66±1.32 years, with a sex ratio of 1.25. The history of the patients found that 36.11 had already been in contact with a COVID-19 positive subject, of which 8 or 22 had close relatives treated for COVID-19 and 5 had been with classmates treated for COVID-19. Fever and physical asthenia, runny nose and throat pain were respectively found in 58.33%, 50% and 30.55% of patients with irritability in 25%. Asymptomatic children were 30.55%. The diagnosis was confirmed after a positive RT-PCR test. Thoracic computed tomography (CT) scan was normal in 80.55% of the children. They were given mostly azithromycin 15mg/kg, zinc and chloroquine sulfate 5mg/kg. The mean age of the patients observed was 9.66 years, with a sex ratio of 1.25. The history of the patients found that 36.11 had already been in contact with a COVID-19 positive subject, of which 8 or 22 had close relatives treated for COVID-19 and 5 had been with classmates treated for COVID-19. Fever and physical asthenia, runny nose and throat pain were respectively found in 58.33%, 50% and 30.55% of patients with irritability in 25%. Asymptomatic children were 30.55%. The diagnosis was confirmed after a positive RT-PCR test. Thoracic computed tomography (CT) scan was normal in 80.55% of the children. They were given mostly azithromycin 15mg/kg, zinc and chloroquine sulfate 5mg/kg.

## Introduction

Faced with an unknown virus, the concerns and preoccupations of health actors in all fields are growing. The International Committee on Virus Taxonomy has defined SARS-CoV-2 and its associated disease as Coronavirus Disease 2019 (COVID-19) [1]. Given its rapid spread worldwide, the World Health Organization (WHO) declared the disease at COVID-19 as a pandemic on March 11 [2]. To date, there have been 37,575,650 positive cases and 1,077,849 deaths at all ages worldwide [3]. In Guinea, there are 11,188 positive cases with 70 deaths [4], with a current population of 14,515,653. To date in our country, we do not have collective epidemiological and clinical data specific to children. The disease can be found at any age given the highly reported mode of contamination and spread by air. There is no evidence of intrauterine infection caused by vertical transmission [5,6]. Amniotic fluid; umbilical cord blood; and neonatal throat swab samples from COVID-19 infected mothers were negative for COVID-19 [7,8]. In addition, there is increasing evidence of neonatal pneumonia induced by SARS-CoV-2 infection [9]. Some authors including Lu X *et al*. Liu W *et al*. based on case series, suggest that children appear to be less affected than adults [10,11]. Although the authors believe that the clinical features, course and outcome of the disease in children and young adults appear to be significantly less severe than in older people, we must point out that there is a serious lack of data regarding the epidemiological and clinical features of COVID-19 in pediatric subjects. Fever and a mild cough are the most commonly described symptoms at the onset of illness in children [12]. Other clinical features include sore throat, rhinorrhea, sneezing, myalgia, fatigue, diarrhea and vomiting. Diagnosis is based on a combination of clinical and paraclinical arguments (laboratory abnormalities, chest imaging and RT-PCR). Saliva may be an ideal specimen type for the diagnosis of COVID-19 infection in children and its use improves the chances of diagnosis. Based on the results of the antibody test, confirmatory RT-PCR and clinical evaluation, hospital treatment or home isolation measures are initiated, with contact tracing measures as per protocol. Based on observations reported elsewhere in the world, the course of the disease and its outcome in children and young adults appear to be significantly less severe than in older people. This study aims to evaluate the main clinical and evolutionary features of SARS-CoV-2 infection in children aged 0 to 18 years who were suspected and diagnosed for COVID-19 in the pediatric ward of Conakry.

## Methods

This is a retrospective study carried out over a period of 5 months from 01^st^ April to 30^th^ September 2020. The work targeted all children admitted to the Pediatrics Department during the study period and focused on children whose clinical examination (fever, physical asthenia, cough, runny nose) and/or history suspected an SARS-CoV-2 infection (contact with a parent or a COVID-19 positive person). Only children with a positive RT-PCR test were included. The clinical data analyzed were age, gender, COVID-19 contact, comorbidity, pneumonia symptoms and clinical signs of complications (neurological deterioration, tachypnea, and hypoxia). CT features were also evaluated. Children with upper respiratory tract infection (i.e. pharyngeal congestion, sore throat and fever) without radiographic involvement were included in the “mild” symptom category. Children with radiologic signs of “pneumonia” and no complications were classified as “moderate” symptoms. On the other hand, children with a mild or moderate clinical picture, with manifestations suggesting disease progression (i.e. deterioration of neurological status, tachypnea, hypoxia, were considered “severe”. Recovery was considered to be a favorable/satisfactory course. Anonymity with no implication of any potential risk to patients and respect for ethical rules was standard. There was no connection between patients and researchers. Patient records were used as the data source and the analysis was done using STATA/SE version 11.2 software.

## Results

The mean age of the patients observed was 9.66±1.32 years, the male sex was the most represented with a proportion of 55.55% versus 44.44% female and a sex ratio (male to female) equal to 1.25 (Table 1). The history of the patients shows that 13 children (36.11%) had already been in contact with a COVID-19 positive subject, of which 8 (22%) had close relatives (father, mother, siblings) who were confirmed positive, treated and cured or under treatment. Similarly, 5 or 13.88% of the children reported having been in contact with classmates who had been treated for COVID-19. In addition, 23 patients, or 63.88%, were unaware of having been in contact or not with a carrier of the virus (Table 2). All of the children had had malaria in the past. Epilepsy was noted as a co-morbidity in one child, and 4 other children or 11.11% had positive HIV serology. Fever and physical asthenia, cough and runny nose, sore throat were respectively found in 58.33%, 50%, and 30.55% of the patients. Irritability was noted in 25% of patients and 16.66% reported myalgia. However, almost half, 30.55% of the children studied in this series were asymptomatic on COVID-19 (Table 3). The rapid diagnostic test (RDT) was positive in both symptomatic and non-symptomatic children on COVID-19. The RT-PCR allowed us to confirm the diagnosis of COVID-19 infection in all patients (Table 4) even though only 94.44% presented a non-specific inflammatory syndrome on biological examination. Similarly, lymphocyte and monocyte blood levels were significantly elevated in all children (Table 5). Chest CT scan revealed frosted glass opacities in 19.44% and Kerley's line in 11.11% of patients. Also noteworthy was the normality of the chest CT scan in 80.55% of patients (Table 5). All patients were treated with azithromycin and zinc; 34 or 94.44% benefited from chloroquine sulfate and in the 5.55% HIV positive patients, we had maintained antiviral treatment. The evolution was marked by the healing of all the children studied in this series (Table 6).

**Table 1:** repairing children based on symptoms and admission information

		< 28 days	28 days - 4 years	5 - 9 years	10 - 14 years	15 - 18 years	Staff
**Number of children admitted to the service**	**Male**	0	1	9	5	5	20
	**Female**	0	3	7	4	2	16
	**Total**	0	4	16	9	7	36
**Average age (year)**	9.66						
**Sex ratio**	1.25						
**Symptoms of admission (list)**	Fever, physical asthenia						
	Cough, nasal discharge						
	Sore throat						
	Cephalalgia						
	Irritability, reduced intake						
	Myalgia						
**Intake symptoms (dominant)**	Fever, physical asthenia						
	Cough, nasal discharge						
**Children with symptoms of COVID-19 at admission**	**Male**	0	0	4	7	3	14
	**Female**	0	1	5	3	2	11
	**Total**	0	1	9	10	5	25

**Table 2:** patient distribution by history and diagnosis

	Positive child at COVID-19	Child negative at COVID-19	Total
**Notion of patient contact COVID-19**	13	0	36.11
**Contact with relative of family treated for COVID-19**	8		22
**Contact with classmate treated for COVID-19**	5		13.88
**Unknown exposure**	23	0	63.88
**Total**	36	0	36

**Table 3:** repair of children according to the frequency of clinical and therapeutic data

Clinical and therapeutic data		Staff (n)	Percentage (%)
**Comorbid**	Epilepsy	1	2,77
	HIV-positive on treatment	4	11.11
	Malaria	36	100
**Signs and symptoms**	Fever, physical asthenia	21	58.33
	Cough, nasal discharge	18	50
	Headache, sore throat	11	30.55
	Irritability/cry, reduced intake	9	25
	Myalgia	6	16.66
	Asymptomatic	11	30.55
**Therapeutic options**	Chloroquine sulphate	34	94.44
	Azithromycin + zinc	36	100
	Antiretroviral	2	5.55

**Table 4:** distribution of children by disease expression and diagnostic test

		Positive		Total
		**Symptomatic at COVID-19**	**Asymptomatic at COVID-19**	
**Diagnostic test**	**RT-PCR**	25	11	36
	**TDR**	13	23	36

**Table 5:** distribution of children according to biological and imaginary data

Paraclinical features		Staff (n)	Percentage (%)
**Biological data**	High C-reactive protein and sedimentation rate	34	94.44
	High lymphocyte count (66.2 - 76%)	36	100
	High monocytes count (10.6 - 14.6%)	36	100
**Chest CT scan and its Characteristics of the lesions**	Normal	29	80.55
	Frosted glass opacification	7	19.44
	Kerley line	4	11.11

**Table 6:** post-therapeutic issues

	Heal	Death	Total
**Male**	20	0	55.55
**Female**	16	0	44.44
**Total**	36	0	100

## Discussion

A recent work by Ludvigsson F. Jonas [13], suggests that infection with CA-SARS-CoV-2 occurs in children, but appears to have a lower incidence, a milder course and a better prognosis in children. Our observation of a mean age of 9.66 years and a sex ratio=1.25 is consistent with the findings of Balasubramanian S *et al*. [14] which exposes the occurrence of the disease in children and at the same time explains why children are less affected than the elderly. The effectiveness of the immune system in young children could be a determining factor in the rarity and benignity of the disease in these children. The gender of the patient does not seem to have an interest in the contamination of the disease. All the male predominance observed in our study could be the consequence of the inclusive character and ignorance of attitudes, guided by a carefree adolescence of young boys.

The history of the patients, which revealed that thirteen (13) had already been in contact with a subject diagnosed and cured for COVID-19, also revealed that 61.53% of these children were probably contaminated by the parents (treated and cured for COVID-19) although the initial RT-PCR test of the children was negative. Furthermore, since a person cured of the disease is no longer a subject of contamination unless re-infected, we consider that these children were probably contaminated at the same time as their parents even though they had not initially presented clinical and paraclinical arguments in favor of COVID-19 infection. If this is the case, it is agreed that there is sometimes a delay in clinical and paraclinical expression of the disease in children. Also, 38.46% of children who have been in contact with a carrier subject have probably been contaminated in a school setting given the notion of contact with a classmate who was followed for COVID-19. Because children are younger and immature, they cannot formally comply with the prescribed barrier measures. Supervisors should increase vigilance and insist on observation and isolation of all children in the event of a positive result from one of the classmates. Several RT-PCR tests should be performed and each child's chart should be checked. Dong *et al*. study of 2143 children identified by laboratory tests using a combination of clinical symptoms and exposure status revealed that 34.1% had laboratory-confirmed disease, while the remainder had clinically suspected disease [15]. Contrary to our observation, all children were diagnosed positive on the basis of the RT-PCR laboratory test. However, our clinical observations were consistent with the typical symptoms of acute respiratory infections and included fever, physical asthenia, cough, sore throat, sneezing, myalgia. The proportion of asymptomatic children found in the literature [14] and observed in our study, extinguishes the thesis of a milder evolution of the disease and a better prognosis in children. However, taking into account the results of some authors who report that even asymptomatic subjects can be contagious [16,17], we believe that this state of affairs would constitute a circle. In our circles, people do not go to the hospital to be screened, but rather to be treated when they experience symptoms. In spite of the 2019 coronavirus health crisis we are currently experiencing, very few people deliberately go to the hospital to get tested. In fact, some people do not believe the disease exists, otherwise for those who do, the disease would not have an impact on the health of people in black Africa. These resilient situations delay diagnosis and therefore increase the risk of contamination and spread of the disease in all sectors, even in schools.

The occurrence of infection and its clinical expression in the four (4) children with HIV, as well as the asymptomatic children (30.55%) despite a positive RT-PCR test, indicates the involvement of immunity in the pathophysiology of the disease. None of our patients presented severe symptoms but only 80.55% had mild symptoms and 19.44% presented moderate symptoms. Almost all children (94.44%) presented a non-specific inflammatory syndrome on biological examination. This could be considered biological evidence of the storm cytokines marker of inflammation of the systems. The RT-PCR test aided the diagnosis. The chest CT scan carried out in all children provided some illustrations of the evolution of the disease. Chloroquine sulfate in application in our country was administered to almost all children with an indication at a dosage of 5mg/kg. On the other hand, children living with HIV on antiretroviral drugs did not benefit from chloroquine adjustment, except for azythromycin, and the rest of the treatment was symptomatic and then a strictly adhered to diet. Although several published studies denounce the ineffectiveness of the molecule in the treatment of COVID-19 infection, in our context, it is attributed to the cure of a plethoric number of patients of all ages, hence the use with respect to the prescribed precautions especially in small and large children. The evolution has been marked by the cure of all children. The lack of collective epidemiological and clinical data relating exclusively to children in our country was the main limitation in this study. A national study of pediatric SARS-CoV-2 infection in the pediatric setting would provide a better picture and would likely allow us to learn more about the epidemiology and collect new data. Similarly, a massive screening in schools across the country could be an asset in our fight to eradicate the disease.

## Conclusion

This study identified 30.55% of children who were asymptomatic on COVID-19 and the rest with mild to moderate symptoms. The evolution was satisfactory in all patients observed, even in those with underlying viral infection. The delayed onset or even absence of symptoms in children suggests an increased risk of contamination and spread because diagnosis is sometimes late and therefore management is delayed. In cases where there is a close relative (father, mother, siblings) who is positive for the disease, we propose immediate observation of the children close to him/her, even if there are no clinical or paraclinical arguments in favor of the disease in the child. The identification of children with co-morbidities and their strict and rigorous management should be a central concern for clinicians.

### What is known about this topic

- SARS-CoV-2 infection can be found at any age but remains more common in adults than in children.

### What this study adds

- The severe form of the disease is rare in children, it has not been found at all in this work. The clinical and paraclinical expression of the disease can be late in children. Asymptomatic forms would represent a potential risk of contamination and spread of the disease.
